# Epistemic authority and medical AI: epistemological differences and challenges in medical practice

**DOI:** 10.1007/s11019-025-10306-2

**Published:** 2025-11-01

**Authors:** Angeliki Kerasidou, Charalampia Xaroula Kerasidou

**Affiliations:** Ethox Centre, Nuffield Department of Population Health, Big Data Institute, Li Ka Shing Centre for Health Information and Discovery Oxford, University of Oxford, Oxford, UK

**Keywords:** Artificial intelligence, Epistemic authority, Accuracy, Medical practice, Medical AI, Epistemic technology

## Abstract

Recent and ongoing advances in medical AI promise to revolutionise medicine by improving the accuracy, speed, and efficiency of clinical care. These promises are responses to the continuous quest of modern medicine to eliminate uncertainty and find answers to crucial questions of diagnosis, prognosis and treatment, while the impressive reported results of medical AI have raised the question of whether medical AI can be perceived as an epistemic authority that challenges the authority of doctors. In this paper, we examine this question by approaching it from the standpoint of what epistemic goods medical AI can offer, or else, what medical AI can claim to “know”. Using Popowicz’ account of epistemic authority in medical practice, which he locates in the scientific method that underpins the practice, we argue that medical AI uses a different scientific method to the one that has given rise and forms the epistemic foundations of traditional western medicine, and this presents a problem. As long as we are seeking not only statistically accurate correlations, but empirically grounded causations in medicine, AI cannot be treated as an epistemic authority in this field. We conclude that until medical practice finds ways to successfully incorporate such epistemological differences, medical AI should submit to the epistemic authority of medical practice and take its place on the long list of important and useful epistemic tools doctors can use to improve the health of patients.

## Introduction

Recent and ongoing advances in medical AI promise to revolutionize medicine by improving the prediction accuracy, speed, and efficiency of clinical care. These promises are responses to the continuous quest of modern medicine to eliminate uncertainty and find answers to crucial questions of diagnosis, prognosis and treatment. Medical AI is joining a long list of technologies used in medicine with the hope of augmenting human doctor capabilities, and improving patient outcomes by increasing accuracy and eliminating human error from medical practice (Topol 2019).

Some welcome the introduction of AI in the clinical space and are hopeful about the changes it will bring (Graboyes and Topol 2017), whilst others are more sceptical and raise concerns regarding the impact it might have on the role of the clinician and the doctor-patient relationship (McCradden and Kirsch 2023; Funer 2022; Kerasidou 2020; McDougall 2019). One of the main concerns relates to the risk of a new form of paternalism emerging, based on the assumption that medical AI, because of its reported superior performance, would replace clinicians as the new epistemic authority in medicine (Chockley and Emanuel 2016; McDougall 2019). Reassurances that the role of AI in the clinical space would be to assist rather than replace doctors, and proposals that the relationship between humans and AI should be collaborative (Sezgin 2023; Lorenzini et al. 2023; Krittanawong 2018) can go some way to address these concerns. Furthermore, reports that claims of AI’s ability to outperform clinicians are overstated (Drogt et al. 2024; Aggarwal et al. 2021; Nagendran et al. 2020) suggest that concerns regarding AI tools challenging the epistemic authority of clinician’s might be only theoretical, and that they might not necessarily manifest on the ground. As some empirical studies demonstrate, healthcare professionals are wary and resistant to the idea of handing over authority to AI tools in clinical care (Dlugatch et al. 2024; Van Cauwenberge et al. 2022). However, the recent approval by the National Institute of Health and Care Excellence (NICE) in the UK of an AI skin cancer detection system that can also be used autonomously,^1^ as well as ongoing questions regarding the implementation of AI tools in the clinical space, including concerns about accountability and disagreement (Grote and Berens 2020; Lang 2022; Kempt et al. 2023), suggest that ideations of AI tools as epistemically comparable to human healthcare professionals persist.

In this paper, we seek to examine whether AI could be seen as some kind of epistemic authority in the clinical context not by examining what kind of agent AI is, but by asking the question of what can AI claim to “know”. Our paper builds on existing analyses regarding the epistemic ontology and role of AI (Alvarado 2023; Ferrario et al. 2024), and asks what kind of epistemic offerings and contribution AI tools can make to the clinical space. We start by locating AI within the long history of traditional medicine that uses technologies of knowledge to improve clinical care by enhancing accuracy and eliminating uncertainty. We then introduce Dylan Mirek Popowicz’s account of epistemic authority (2021) as located in the scientific method that underpins medical practice. We argue that what AI, and in particular for this case, what Machine Learning (ML)^2^ “knows” is very different from what a medical practitioner knows, or more specifically what the medical practice’s epistemic aims are. We conclude that in so far as medicine remains a scientific endeavour that seeks to know not only “what is” but also “why it is”, medical AI cannot be seen as the epistemic authority in the clinical context. We suggest that there is a clear need to rethink the way medical doctors are trained today, as a way of utilising the epistemic goods/insights offered by medical AI, and particularly ML.

## Medical uncertainty and technologies of knowledge

Medicine has always relied on technologies, including tools, frameworks and processes to improve diagnostic and treatment accuracy, minimise uncertainty and improve patient outcomes. From simple technologies like the stethoscope that augmented physician’s ability to listen to the hearts, lungs and intestines of their patients to more complex ones like X-ray machines and MRI scanners that augmented doctor’s sight, technologies have always been part of western medicine. Knowledge frameworks such as evidence-based medicine (EBM) have also been developed to decrease uncertainty and improve consistency and quality of care. EBM sought to augment not the physical but the cognitive abilities of healthcare professionals by establishing a hierarchy of evidence upon which medical decisions should be based. It placed randomised controlled trials (RCTs) and meta-analyses at the top of the epistemic hierarchy, and medical intuition and mechanistic reasoning at the bottom, introducing a new way medicine was practiced, as well as a new understanding of the epistemic qualities and abilities of medical authority (Sackett et al. 1996). It achieved this by establishing a new way of training medical professionals, moving the practice further away from empiricism, and focusing more on ‘how to perform and interpret scientific evidence in terms of assessment of credibility, critical appraisal of the results, and integration of scientific evidence in the everyday work’ (Svantesson 2019: 4).

Medical AI, with its ability to process vast amounts of data for more timely and precise diagnoses, prognoses and treatment plans, is a further step into the technology-supported quest to eliminate uncertainty in medicine. However, as Alvarado notes, AI differs significantly from other epistemic technologies that seek to enhance and augment users’ abilities (Alvarado 2023). AI, including medical AI, is designed, developed and deployed: for use in an epistemic context, to deal with epistemic content, and to carry out epistemic operations on such content (Alvarado 2023). It is particularly the two latter characteristics noted by Alvarado, namely the fact that AI deals with epistemic content and carries out epistemic operations, that distinguishes AI from other technologies, that are designed, developed and deployed to help with the acquisition of knowledge (e.g. stethoscope) or the performance of epistemic tasks (e.g. EBM framework for clinical decision making). Ferrario et al. (2024) add a further characteristic that differentiate AI tools from other epistemic technologies. It is AI’s specific ontology as a computational system that is able to learn from the input data and is not fully determined by its designer’s knowledge and intentions, they argue, that make these systems stand out from other epistemic technologies used in medicine (Ferrario et al. 2024).

It is these aforementioned specific abilities of medical AI that give rise to concerns regarding AI replacing doctors as practitioners and as epistemic authorities, as it is assumed that AI tools can know what a human doctor knows, or aims to know. It has been argued, for example, that medical AI tools have medical expertise analogous to that of their human counterparts (Grote and Berens 2020). In so far as AI tools can outperform doctors in their epistemic tasks (e.g. diagnosis), some have argued, human doctors have an obligation to defer to AI and treat it as an epistemic authority (Bjerring and Busch 2021). Others however, have rejected claims that AI tools should be conceptualised as epistemic agents that can hold expertise or authority in a specific domain (see Ferrario et al. 2024). For example, Ferrario et al. (2024) suggest that AI tools do not pass the threshold for epistemic agency because they do not have the kind of relationship with understanding that is expected of epistemic agents. Understanding requires the ability to recognise that something is true and appreciate the significance of truth, and also have the ability to act on true knowledge. Since AI tools lack all of these abilities, they cannot qualify as epistemic agents (Ferrario et al. 2024).

However, even if we accept that AI tools cannot be epistemic agents in the strong sense, there might be reasons to consider medical AI as some kind of epistemic authority to which decision deference would be justified. As mentioned above AI tools are a special type of epistemic technology that does not only operate within an epistemic context but also carries out epistemic operations, often with greater degree of accuracy than when the same operations are carried out by humans. If the aim of the epistemic operation is greater accuracy of prediction, having a particularly relationship to and understanding of the truth as required by epistemic agents, might not be significant (London 2019). Perhaps following this logic, what is more important is the ability to make truthful utterances in the form of accurate predictions, as opposed to holding true beliefs which assumes a certain relationship to truth and understanding, especially in situations of disagreement between human doctors and AI (Grote and Berens 2020; Lang 2022; Kempt et al. 2023).

In order to examine whether AI could be said to hold some kind of epistemic authority in the medical context, we turn to Popowicz’s account of epistemic authority. We use his account as it addresses this question of authority specifically within the domain of medicine. We approach the question of authority from the standpoint of what it is that AI can claim to “know”.

### Scientific method as an epistemic authority

An epistemic authority is someone that based on their superior epistemic position have the authority to tell others what to believe or how to behave within a specific epistemic context (Popowicz 2021). It has been argued that recognizing someone as an epistemic authority commits one to taking their (epistemic authority) beliefs for x as one’s beliefs, and dismiss any contrary beliefs one might have (Zagzebski 2012). This normative implication of preemptively replacing one’s beliefs with someone else’s beliefs based on acceptance of their authority in a specific domain has raised objections as being too demanding. Although some have argued, for example, that preemption is never epistemically justified (Lackey 2016, 2018; Hauswald 2021), it still makes intuitive sense to believe that there will be circumstances in which deferring to one we accept as being an authority and of possessing superior knowledge to us in a specific domain would be the reasonable thing to do (Wright 2016; Jäger 2016; Dormandy 2018; Stewart 2020).

One of the theorists examining the concept of epistemic authority within the specific context of medicine is Popowicz. In his two essays, *“Doctor Knows Best”: On the Epistemic Authority of the Medical Practitioner* (2021) and *The Epistemic Authority of Practice* (2024), Popowicz assesses Zagzebski’s normative position of preemption, and suggests that it is not suitable for a non-domain specific context that is medicine and clinical care. According to him, it is not ‘first-order beliefs’ that differentiate an authority from a non-authority, but rather the possession of ‘higher-order issues’ (2024). This means that ‘an epistemic authority can tell me what is and is not a good reason to believe something, what the state of evidence is in relation to a certain question, or how one should go about answering a question in the relevant domain of inquiry’ (Popowicz 2021: 6). Doctors, he argues, are tasked with answering questions such as what is the right treatment for this particular patient, relative to patient’s situation and specific condition of health. These are not domain-specific questions but rather they are subjective and value-laden questions which require input from other epistemic agents, such as the patient. The role of the doctor, as the epistemic authority, is not to provide the patient with answers *vis a vis* true beliefs that should be taken at face value and replace the patients’ own beliefs and reasons. Rather, the doctor is tasked with helping the patient understand what constitutes good reasons upon which she can form her beliefs and henceforth action (i.e. accept the treatment, take the medication, undertake the diagnostic test, etc.) (Popowicz 2021, 2024).

For Popowicz, a doctor’s ability to help a patient make sense of their situation comes from their ability to use a specific scientific method and practice to process information and provide reasons. This way he locates epistemic authority away from the agent and on to the scientific method, and shifts the focus from the “know-what” and on to the “know-how”. He writes,

The ultimate normative “authority” here is the practice itself: if it truly is a good way of getting to the truth, or the best answers in a certain domain of inquiry, then it is what gives any opinion or view an authoritative power. Some opinion is authoritative not because it is expressed or believed by some individual S, but because S’s uttering or believing it is the result of an epistemic practice, which suggests that the opinion is true/evidentially warranted, and so on. […] I identify someone as an epistemic authority not because they somehow possess an extra special acquaintance with the truth as an individual, but because they are better accustomed to a certain mode of inquiry—a methodology I identify as an epistemically good one—because they have the tacit and explicit knowledge of how that inquiry is to be done, and how it works. (Popowicz 2021: 17)

According to Popowicz, the reason we engage with epistemic authorities, particularly in the medical context, is not only because we seek to be closer to the truth but because we want to make sense of how beliefs and reasons we might hold also relate to other reasons and evidence we might acquire to help us form an epistemic stance (namely, accept something as true and act on it). Therefore, the reason we consider doctors as epistemic authorities in medicine is not because they have a special relationship to the truth but because they ‘possess the appropriate know-how to participate in an epistemic practice that we deem epistemically fruitful’ (Popowicz 2021: 5).

The main epistemic practice in traditional western medicine today is EBM. Although, its introduction in the 1990 s was hailed as a significant epistemic shift, as White and Willis note, EBM operates within the same ‘positivistic, mechanistic and reductionist scientific model’ that underpinned the Baconian scientific method of the 16th century, Koch’s bacteriological principles of late 19th century, and the application of laboratory-based medicine in clinical practice by Flexner of the 20th century (2002: 8). In other words, western traditional medicine as a practice has been built and is still based on a scientific model that uses empirical data to form and test hypotheses with the aim of deriving universal truths. The introduction of AI challenges the foundations of this practice.

## AI as an epistemological challenge for medical practice

If we accept Popowicz’s account of epistemic authority in medicine as being located in the scientific method rather than in the agent herself, the introduction of AI in the medical context introduces an important epistemological challenge. As Grote and Berens argue, ‘both the clinician and the machine learning algorithm might be perceived as experts of sorts. Yet, they have been trained differently and they reason in very distinct ways’ (2000: 207, see also Tikhomirov et al. 2024). Therefore, it is important to understand what are the epistemic goods AI can offer generally, and in clinical practice specifically.

While the field of data science appears to imitate the fundamentals of conventional science and to operate within the same rationalist, universalist and relatively static assumptions of objective knowledge (Birhane 2021; McQuillan 2022), data science has developed into a new field of enquiry, or a new kind of science, with theorists attempting to unify its epistemological foundations under a coherent framework (Maruyama 2019, Desai et al. 2022). According to the traditional western theory-centric conception of science, scientific knowledge consists of justified true beliefs about the world which are obtained through empirical methods. Based on a specific gnostic relation between hypothesis and evidence, in the classical conception of scientific method, hypotheses are derived from existing theories and then confirmed (or not) through empirical methods such as experiments (Desai et al. 2022). Data science, on the other hand, introduces a new *agnostic* approach to science. As Desai et al. explain,

Here, scientific knowledge can be generated, and mathematical and data-scientific methods deployed without prior knowledge or understanding of phenomena or their interrelations. A putatively agnostic science is one where experiments are in some sense “blindly” performed, and large amounts of data amassed (2022: 469).

From these ‘large amounts of data’, AI algorithms retrospectively seek to establish correlations rather than causations. Whereas in traditional science the trajectory is to move from empirical data to universal laws, in data science the move is from data to contextual laws that have no other application beyond the specific empirical context within which they have been developed. As Munn et al. explain, an AI model ‘does not “know” of any reality outside of itself’ against which it can verify its outputs (2024): 2761). In that sense, ‘AI is not realist but instrumentalist’ (McQuillan 2022: 48). It does not try to understand and model the actual dynamics of a system, it only models the world to get “accurate” enough outputs that it has no way of verifying if they are, indeed, true. The methods and also the aims of traditional science are very different to that of data science. As Maruyama notes, ‘traditional science aims at infallible knowledge’, whereas ‘data science aims at fallible statistical knowledge’ (2019: 546-7). This means that traditional medicine and medical AI operate within very different scientific and epistemic frameworks.

Having established that medicine as practice and medical AI operate within very different epistemic frameworks, using different methods and having different aims, the question arises of whether it is reasonable to perceive AI as an epistemic authority in the context of today’s medical practice. It could be argued that medicine as a scientific practice is theory-centric, however, clinical care on the ground differs significantly, and it is actually closer to AI’s truly empirical and atheoretical epistemic nature. For example, as London (2019) argues, the mechanisms by which an ML tool surpasses expert doctors’ skills are as opaque as the reasons why Lithium works as a mood stabiliser. Indeed, in medical practice, the empirical findings and inherited clinical culture can sometimes tramp the lack of knowledge of the underlying causal system. In other words, doctors’ recommendations can be similarly opaque, associationist and atheoretical as the outputs of the AI system, as London argues (2019). The inability to explain how results are produced, London (2019) continues, should be less important than the ability to produce such results. In other words, what matters on the ground is actually “what is”, rather than “why it is”.

However, pointing out that often human doctors practice medicine even when causation is not established, does not necessarily mean that we ought to abandon the practice all together. To put it differently, while we might not know exactly how the mind of doctors’ work when reaching their diagnosis, modern medicine has built an institutional system - underpinned by the traditional scientific method - of educational training, scientific and logical procedures and clinical processes and practices within which healthcare professionals are expected to operate. And while acknowledging that causal gaps remain in medical knowledge, processes such as RCTs are one of the ways that the discipline tries to address these gaps (Hariton and Locascio 2018) rather than merely accept them. In that sense, medicine keeps trying to eliminate uncertainty not merely by collecting more observations and identifying correlations, but by continuing its efforts to establish causations, since for traditional medicine, and for the epistemic agents who participate in it (healthcare professionals, patients), what matters is not only that something is but also why it is. Doing otherwise, as Alvarado argues (2022), and merely accepting something *because it works*, regardless of its relationship to truth, might be based on pragmatic reasons but not on epistemic ones.

Alternatively, in order for medical AI to be accepted as an epistemic authority to which doctors and patients ought to defer, this would presuppose a decision that it is pragmatic reasons that ought to prevail in clinical practice rather than epistemic ones. Such a stance would mark a true epistemological shift in clinical care (White and Willis 2002), as it would move medical practice away from the positivist and universalist scientific framework and into a truly empirical and atheoretical one. However, whether such a shift ought to take place is both a philosophical (e.g. how should we understand medical knowledge and evidence? How will such a shift affect the epistemic and normative doctor-patient relationship?) and empirical (e.g. would patients be satisfied with diagnoses, prognoses and treatment plans that are only and purely empirically derived?) question that requires further investigation (see, for example, Funer 2022; Tikhomirov et al. 2024).

Raising issues such as the above, is not to deny the impressive recent advancements of AI and its potential benefits for healthcare, but a call to better understand what kind of epistemic goods AI can offer to medical practice, and whether and how it can improve care on the ground (Tikhomirov et al. 2024; Samhammer et al. 2022; Goulenok et al. 2025). Recent studies and reports of doctors performing badly when AI is involved, of requiring greater epistemic reliability before they incorporate AI tools in their practice, or of patients not feeling valued when directed to AI, or of them not following AI advice (Formosa et al. 2022; Radionova et al. 2023; Dlugatch et al. 2024) demonstrate that providing an answer to these questions is not straightforward.

If we accept Popowicz’s account of epistemic authority as being located in the scientific theory that supports a practice, then the fact that AI relies on a different scientific model, presents a problem. As long as we are seeking not only statistically accurate correlations, but empirically grounded causations in medicine, AI cannot be treated as an epistemic authority in this field. However, acknowledging the epistemological differences between the scientific foundations of medicine as a practice and of data science and AI, sensitises us to the ways that the introduction of this new technology disrupts existing practices, and as such, prompts us to ask questions such as what additional elements should medical training incorporate in order to equip practitioners with the right knowledge and skill to harness the advances in AI for patient care (Giordano et al. 2021; see also Liu et al. 2022; Grote and Berens 2020; Futoma et al. 2020), and also, what is needed in order for medical AI to be successfully incorporated into clinical practice, without undermining epistemic authority but by extending it.

## Conclusion

In this paper, we sought to examine the question of whether medical AI can be perceived as an epistemic authority, even in a weak sense, by approaching the question from the standpoint of what epistemic goods it can offer, or else, what it can claim to “know”. We used Popowicz’s account of epistemic authority in medical practice. According to Popowicz, epistemic authority in medicine is located in the scientific method that underpins the practice. We argued that medical AI operates within a different scientific framework to the one that has given rise and forms the epistemic foundation of traditional western medicine. Whilst medical practice is situated within a positivist and universalist scientific framework that uses empirical data to establish theory, AI originates from a scientific framework that is atheoretical and committed to establishing statistical correlations rather than causal links. This significant epistemological difference between the scientific underpinnings of traditional western medicine and AI suggest that medical AI cannot be perceived as an epistemic authority to which one could defer clinical decisions. Whilst it is possible to assume that a scientific practice that seeks to establish “what is”, rather than “why it is” might come to prevail in medicine, such a change will mark a significant epistemic shift in this field. Yet, and until this happens, medical AI should submit to the epistemic authority of medical practice and take its place on the long list of important and useful epistemic tools doctors can use to improve the health of patients.

## Footnotes

1
https://www.nice.org.uk/news/articles/ai-skin-cancer-detection-system-gets-green-light-for-conditional-nhs-use (last accessed 01 Aug 2025).

2 We use AI as the prevailing umbrella term to refer to computational techniques that can be used to make machines complete tasks in a way that would be considered intelligent were they to be completed by a human. Here we specifically refer to Machine Learning (ML) which is a particular, and currently the most prevalent, type of AI which involves computers ‘learning’ without specific instructions, doing so by using algorithms to analyse and draw inferences from data. We use the two terms interchangeably.
